# Opportunities and Challenges to Improve Postpartum Care: Payors’ and Purchasers’ Perspectives in California

**DOI:** 10.1089/whr.2024.0198

**Published:** 2025-04-21

**Authors:** Lindsay Parham, Renee Clarke, MariaDelSol De Ornelas, Sabrina Li, Sylvia Guendelman

**Affiliations:** School of Public Health, The Wallace Center for Maternal, Child, and Adolescent Health at the University of California, Berkeley, California, USA.

**Keywords:** payors, purchasers, postpartum care, postpartum, reimbursement, insurance

## Abstract

**Introduction:**

U.S. maternal mortality rates are two to three times higher than other high-income countries, with most deaths occurring postpartum. Fragmented care, exacerbated by health insurance gaps and workforce shortages, underscores systemic deficiencies. Although patients’ and clinicians’ perspectives are well-documented, little is known about payors’ and purchasers’ perspectives. Given their influence in coverage decisions, payment rates, and service reimbursement, the objective was to explore their perspectives and identify challenges and opportunities in improving postpartum care in California, currently engaged in reshaping maternal health pathways.

**Methods:**

We conducted a qualitative study using semi-structured interviews with high-level administrators from major California health insurance providers and purchasers between June and October 2023. Participants, recruited through professional connections, were selected through purposive sampling based on their involvement in maternal and child health coverage decisions. A hybrid inductive–deductive approach was employed to identify major themes.

**Results:**

Participants (*n* = 11) identified barriers including limited insurance coverage, lack of clinical provider incentives, reimbursement concerns, and misaligned measures and metrics. Opportunities to improve postpartum care focused on visit timing and frequency, alternative payment models, and improving continuity of care between birth and the transition to primary care.

**Conclusions:**

Insurance payors and purchasers identified postpartum care barriers and suggested solutions well-supported by the literature. These solutions—including reimagining global bundle payment models, updating Healthcare Effectiveness Data and Information Set measures, and promoting dyadic models—could address barriers, improve outcomes, and inform California’s ongoing maternal health transformation and those happening around the United States.

## Introduction

Maternal health policy and clinical reforms have primarily focused on the antepartum and intrapartum periods, overlooking the postpartum period.^1^ In recent years, health care professionals, policymakers, and researchers have shifted attention to the postpartum period (the “fourth trimester”),^2^ recognizing its wide-ranging impact on maternal health outcomes. In 2022, the U.S. maternal mortality rate reached 22.3 deaths per 100,000 live births, far surpassing other high-income countries.^3^ In 2020, the Centers for Disease Control and Prevention (CDC) reported that 63% of pregnancy-related deaths occurred in the postpartum period, with 80% deemed preventable.^4^ Deep racial disparities further exacerbate this issue, with Black birthing individuals being three times more likely to die during pregnancy or postpartum than White individuals.^5,6^ These findings underscore the urgent need to improve postpartum care in the United States, particularly for marginalized populations.

Postpartum care in the United States traditionally consists of a single medical visit approximately 6 weeks after birth.^7^ However, clinicians, patients, and experts increasingly view this traditional model as insufficient for addressing the critical health concerns of new parents and their infants.^7–9^ Furthermore, systemic barriers have contributed to high attrition rates for these visits.^10,11^ Several studies have identified what patients and clinicians consider key barriers to improving postpartum care quality and access. Medicaid patients report obstacles in obtaining pregnancy-related Medicaid coverage, finding clinical providers who accept Medicaid, maintaining clinician continuity, and securing transportation and childcare to attend postpartum visits.^12^ In addition, unmet material needs, insufficient resources, and insurance coverage discontinuity hinder optimal postpartum care.^12,13^ A 2022 qualitative study further revealed that many new parents struggled with breastfeeding, emotional and mental health needs, mobilizing support, and lacked adequate education.^14^

Likewise, literature on clinicians’ experiences identifies several administrative and payment-based hurdles. Clinicians reported difficulties in scheduling appointments,^15^ lack of continuity between clinicians,^16^ knowledge gaps, trouble accessing referrals, ambiguity in clinician roles,^15^ and the need for improved care coordination, particularly regarding continuity of care and health coverage.^16,17^ These needs could be addressed through new care delivery models such as telehealth visits, “centering” group visits, home visits, doulas, and peer support groups.^14^ However, most insurance plans do not cover these innovations, limiting their availability nationwide.^18–20^

Professional organizations have worked to address the clinical gaps in postpartum care. In 2018, the American College of Obstetrics and Gynecology (ACOG) recommended that postpartum care include an initial assessment with obstetric care providers within the first 3 weeks postpartum, followed by ongoing care as needed, and a comprehensive visit by 12 weeks postpartum.^7^ Despite these recommendations, evidence suggests that many organizations, private practices, and hospitals have yet to adopt them.^15,21^

Understanding the perspectives of insurance providers and purchasers is crucial for successfully implementing clinical recommendations such as ACOG’s and improving postpartum care. Insurance providers play a pivotal role in shaping health care delivery in the United States. They decide which services are “covered” and set reimbursement rates for clinical providers, determining what is accessible for members and what patients pay out-of-pocket.^22^ Through these funding decisions, health plans define and regulate what type of postpartum care is available.^23^ Despite their critical role in shaping access to and quality of postpartum care, insurers’ perspectives have been largely underexplored, creating a significant gap in understanding how their decisions influence care delivery and maternal health outcomes.

This study explores the perspectives of insurance payors and purchasers regarding the barriers and opportunities for innovating postpartum care, focusing on those operating in California for two key reasons: (1) the state’s health care system impacts 1 in 10 U.S. births and (2) the state is a leader in efforts to improve maternal health outcomes.^24^ Recent policy innovations include guaranteed income for pregnant individuals at high risk of preterm birth,^25^ funding for culturally congruent doulas and community health care workers (CHWs),^26,27^ and research to end preventable maternal morbidity, mortality, and racial disparities.^28^ Currently, California’s Department of Healthcare Services (DHCS) is working with the state’s Medicaid operator (Medi-Cal) to implement new birthing pathways aimed at—among many goals—improving and supporting better postpartum care.^30^ Focusing on California allows us to identify opportunities and barriers for insurers at the forefront of innovative maternal care policy. For states following similar paths, these findings offer valuable insights for future public health campaigns to innovate postpartum care.

## Methods

We conducted a qualitative study using semi-structured interviews with high-level administrators of major commercial health insurers, Medi-Cal programs, health insurance purchasers, and a pension plan in California. We recruited participants through professional connections and used purposive sampling to identify key stakeholders involved in high-level maternal and child health insurance decision-making. We then focused on people involved in decision-making related to maternal and/or postpartum care. Recruitment took place between April and October 2023. The study team (L.P. and S.G.) sent recruitment emails containing key information and invitations to participate in the study. The study team sought support from the California Health Benefits Review Program (CHBRP), which evaluates the “medical effectiveness, cost impact, and public health impact of bills related to health insurance benefit.” CHBRP regularly surveys insurers about their coverage policies and leveraged its professional connections to guide the team to key organizations to contact about postpartum care.

**FIG. 1.**
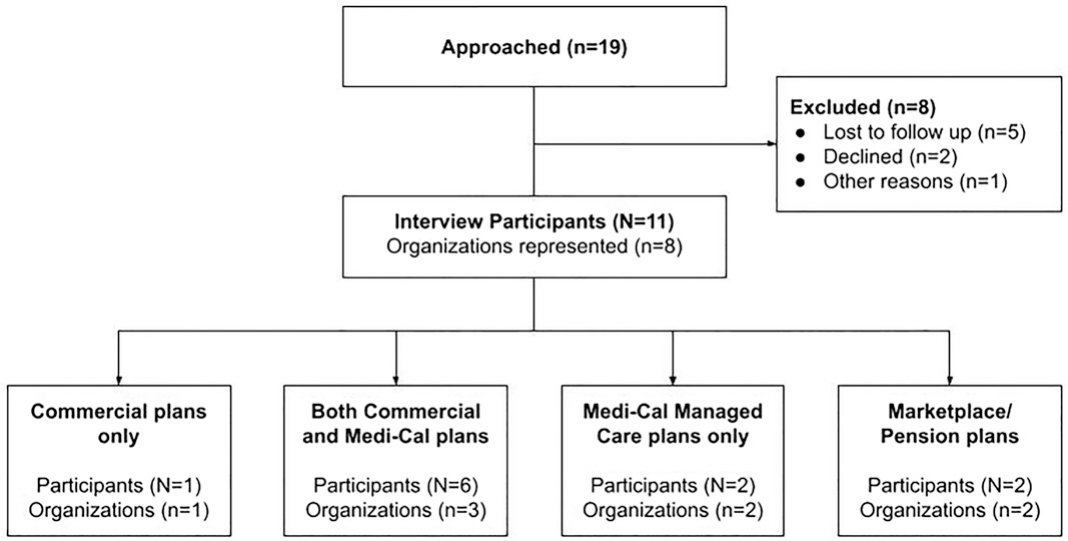
Participants and their organizations.

We used a convenience sample to select 19 key administrative health care leaders from different coverage areas with large market shares in California. Of these, five were lost to follow-up emails, two declined on the day of the interview (one due to conflicts of interest, one based on advice from their legal department), and one missed a pair interview (illness). Ultimately, 11 participants consented and completed interviews with the research team. Between June and October 2023, nine interviews were conducted by the study team (L.P., S.G., M.D.O., and R.C.) with *n* = 11 participants from eight organizations (Fig. 1).

The interviews used a semi-structured guide to capture the adequacy of postpartum care in California, standard practices and reimbursement coverage, and views on innovative approaches to enhance the postpartum experience. These topics were informed by three focus groups conducted during a pilot study with clinicians (*N* = 11), which indicated that insurance coverage and reimbursement rates significantly impact barriers and opportunities for improving postpartum care. Each interview lasted 45–60 minutes, with consent documents and an overview of interview topics provided in advance for the participant’s review. Interviews were conducted via videoconference technology or phone and were digitally recorded to capture audio only. All participants provided oral consent before participation.

Interview recordings were securely stored in UC Berkeley’s cloud storage platform, behind the university’s firewall. Recordings were professionally transcribed and then cleaned by the study team (R.C.) to remove potential identifiers. The coding team (L.P., R.C., and M.D.O.) used the qualitative data analysis software, Dedoose, for analysis. The analytic process used a hybrid inductive–deductive approach.^31^ The study team coded according to a template codebook based on themes identified in the literature and included in the interview guide and deductively identified emergent themes and patterns in the raw data. The analysis proceeded in three stages. First, coders (M.D.O. and L.P.) used a rapid analysis approach on two interview transcriptions, identifying eight key themes related to postpartum care barriers and opportunities. Senior members of the study team met regularly to assess themes and content at this stage. Using a combined iterative approach, analysts (L.P. and R.C.) then applied the codebook to two different transcripts, refining code definitions and removing or consolidating unused codes. The resulting codebook was then applied to all transcripts by the analysis team, with regular meetings to assess coding consistency and resolve discrepancies. A total of 305 excerpts were coded and categorized by theme and subtheme.

This study was approved by the University of California, Berkeley’s institutional review board (ID: 2023-01-15992).

## Results

We interviewed eight organizations in California that were either payors or purchasers: five contracted with Medi-Cal as managed care plans, one was solely commercial, one served as an insurance purchaser, and one established an insurance marketplace. As of December 2023, the five organizations offering Medi-Cal plans we spoke with cover nearly 60% of the Medi-Cal enrollee population, encompassing over 30 counties. Four stakeholders from four commercial insurers interviewed collectively represented 81% of the California insurance marketplace in 2022. In addition, we spoke with medical directors from a large public employee health benefits purchaser and a major insurance marketplace for individuals and small businesses, which combined covers over 3 million enrollees. Several participants self-identified as parents with personal experience with postpartum care in California. CHBRP verified that our recruitment sample included insurers that covered a large majority of California’s insured population (Fig. 1).

Participants were asked about their perspectives on the adequacy of postpartum care in California, standard practices, reimbursement coverage, and innovative approaches to improving the postpartum experience (Table 1). Most (10 of 11) felt the current system lacked sufficient support for new mothers. A respondent from a large commercial insurance purchaser remarked: “I feel like there is insufficient support … our current infrastructure is poorly designed to be able to meet all of the needs that come up in the postpartum period.” A respondent from a Medi-Cal organization described the system as “bare minimum” and “not good enough,” noting that many postpartum visits, especially among underserved patients, are missed.

**Table 1.** Perspective on the Current Postpartum System

#ID	“Is the Current Postpartum System Supportive of Mothers?”
*P11*	“I certainly think that there is support [but] that there is plenty of room to do better … . [i]f you look at the outcomes, there are a lot of *disparities*.”
*P09*	“Well, I think just the *length of follow-up [is an issue]*. I mean *six weeks is just not adequate* … . See, the whole issue is that we’re still laboring under the global OB concept where, as part of the global fee, you’re basically paid for everything that happens during the first six weeks. We really should make that *a separately reimbursable period of time and extend it beyond six weeks*. I mean, this concept that somehow at six weeks all mothers are magically healthy … *[i]t was never based on anything.*”
*P06*	“I wouldn’t say that it’s supportive. I mean, it’s *bare minimum*, And there are many, many postpartum visits that are missed by a lot.”
*P20*	“I think that *there’s a lot more that can be done*. And I know that Medi-Cal has expanded the postpartum care up to a year. And so, I think we should really be *expanding [care] to … a fourth trimester of pregnancy or the postpartum year … . [I]t should be about family-centered care*.”
*P12*	“We’re still not there yet. I will say, the biggest downfall … [f]rom a financial perspective has been the lack of *investment in the Medicaid space*. And this is as a former regulator.”
*P01*	“There are certainly *gaps in care*. One of the things that we’ve been looking at is *mental health in general and access to mental health services …* [w]e’ve been hearing through feedback from our members *… there is a real gap in care in terms of access to mental health services*.”

OB, obstetrical.

After discussing the current system, participants were asked about the maximum number of covered postpartum visits, reimbursement methods used in the postpartum, how visits after 6 weeks postpartum are addressed, and the integration of clinical recommendations into postpartum coverage. Interviews concluded with a discussion about what an “ideal” postpartum care system might look like. Two recurring patterns emerged from these discussions: current barriers to postpartum care delivery and opportunities for improvement.

### Barriers

The research team defined barriers as any obstacle that prevents access to optimal and timely postpartum care. Three thematic areas emerged through this examination: poor transition between postpartum and primary care services, ineffective postpartum reimbursement structures, and concerns about misaligned measures and metrics that define, capture, and reimburse for quality postpartum care (Table 2).

**Table 2.** Payors and Purchasers: Perceived Barriers in Postpartum Care

Themes	Subthemes	Exemplary quotes
Transition of care	Workforce and professional ownership	“… [C]hanging [postpartum care] to 12 months does what? *Who is going to care for the birthing folks?* … I haven’t really heard it yet. I’ve heard an over-reliance on doulas to solve all of our problems. And that makes me feel uncomfortable as a primary care provider, right? *Where are we stepping up to say that we’re going to do things differently or in a more thoughtful way?* Where are our OB/GYN folks saying I’m happy to carry the torch all the way to 12 months? I haven’t quite heard that yet in the landscape.” (P19)
	Care coordination	“I would say just in the *lack of connectivity and incentives just financially* … the provider who is treating on a fee for service based arrangement where it’s just for the services being provided *does not have a shared incentive to ensure that there’s this warm handoff to the outpatient setting* or a practitioner that they (patient) have no relationship with, they’re getting paid purely for the services that are being provided when they’re showing up. There’s no *care coordination* components.” (P12)
Reimbursement	Negative incentives—bundled payments	“The best patient was the one who skipped their postpartum visit because it was like, I’m not getting paid for this anyway. So, it’d be great if they don’t show up. You know, I had *no motivation to encourage patients to even attend their six-week postpartum visit* … [b]ut if I got paid for it, it would be like, yeah, sure call them up … [H]ave them come in twice, three times, whatever … the incentive wasn’t too great. [If] [h]alf of our six-week postpartum patients didn’t show up, great. … So, the *incentives for postpartum care are completely perverse right now. I mean, the reimbursement system is set up to discourage postpartum visits, plain and simple*.” (P09)
	Low fees for OBs/Gyns	“… [n]o, I have seen as a physician that the provider reimbursement definitely lags for postpartum care. *So, they’re trying to increase what we pay for postpartum care*. But if the state does not pay the health plans for services, the health plans are rarely going to pay higher than what the state pays us. Because where do we get it from? We’re a Medi-Cal plan. *So, frankly, the state needs to increase what it pays providers. And specifically, physicians*.” (P08)
Measures and metrics	HEDIS misalignment (negative incentive)	“I would say that a lot of our outreach…[w]ith providers, our phone calls, our texting campaign, our CHW programs, our CBO partnerships, a lot of that is focused on getting the members seen within that [HEDIS] window. So certainly … that aligns pretty well. *But … if it’s within the first seven days, then that doesn’t align with that HEDIS measure*.” (P11)
		*“One of the things that I think needs to kind of catch up is that ACOG is saying the earlier touch bases are important. Yet, we’re still held to these HEDIS metrics … .* That [patients] get what they actually need would be great instead of trying to meet these targets.” (P20)
	Lack of statewide data	“Part of the *problem for health insurance companies is still the big metrics*, especially maternal mortality. Even though the numbers are way too high nationally, for one insurance company, *you can’t track if your interventions are working, because we have so few women. Our populations just aren’t big enough*. You have to look at it at a state level and at a national level.” (P07)
	Lack of cohesive data management	“And *there’s lots of reasons for [not meeting goals] from problems with data*. But some of the problem is that members that we haven’t reached are not getting that care. If we know about the member, they get that care. But [there’s] a lot of *members we never can reach* **….**” (P08)

ACOG, American College of Obstetricians and Gynecologists; CBO, community-based organization; CHW, community health care worker; HEDIS, Healthcare Effectiveness Data and Information Set; OB, obstetrical; OB/GYN, obstetrician/gynecologist.

#### Transition of care

The transition of care is a crucial follow-up period for postnatal patients who experience physical, emotional, and psychological changes, necessitating continual support.^7^ In the current system, most women consult a prenatal or obstetric clinician for their postbirth visit around 4–6 weeks after delivery before transitioning to a primary care clinician.^32^ Study participants noted that if postpartum care extended beyond the 6-week visit to 12 months, as indicated by Medicaid expansion and acknowledged by ACOG, it remains unclear which branch of medicine should “carry the torch” during that period. One participant called on clinician groups to clarify which professional community should assume responsibility for the 12-month postpartum period. Several participants cautioned against placing increased expectations on the already overburdened doula community to fill this gap.^33^

Another subtheme was the lack of care coordination between postpartum and primary care clinicians. Participants described significant gaps in coordination, particularly within fee-for-service models, where clinicians are compensated for each service performed.^34^ Since these models do not reimburse for coordination activities, several participants claimed there is no “shared incentive” for a “warm handoff” between clinicians after 6 weeks postpartum. This often leaves patients under the care of a new practitioner with no established relationship, who may lack specific knowledge of their pregnancy, birth, or postpartum experiences.

#### Reimbursement issues

Respondents frequently identified reimbursement issues as a major barrier to effective postpartum care. Recently, global fee models, which consolidate all perinatal care—prenatal through postpartum—into one bundled payment known as a “global maternity fee,”^19^ have gained popularity.^35^ This payment is typically disbursed at delivery before the postpartum period begins. One private sector respondent described this model as “set up to discourage postpartum visits,” noting that clinicians receive no compensation for care beyond the birth event.

Low reimbursement rates for postpartum care also emerged as a barrier, with participants reporting that Medicaid, including Medi-Cal, and private insurance rates are insufficient for clinicians. Many stressed the need to raise reimbursement to improve care quality and timeliness. One participant noted that low reimbursement rates disproportionately affect patients seeking culturally congruent clinicians, particularly those relying on Medicaid. Federally Qualified Health Centers (FQHCs) were cited as an exception, as their reimbursements reflect “actual” operational costs: FQHCs use the Prospective Payment System, which bundles all services and supplies per visit.^36^ Participants with experience working with FQHCs reported that these higher reimbursements enabled better postpartum care.

#### Misaligned measures and metrics

Health care organizations and payors utilize various metrics to improve patient care, ensure effective practices, and maintain closed-loop accountability. When asked about metrics for postpartum care, three subthemes emerged: misalignment between national health care accountability metrics and professional postpartum care recommendations, a lack of statewide postpartum data, and inadequate data management cohesion.

Many respondents identified the Healthcare Effectiveness Data and Information Set (HEDIS) as a primary accountability measure used by their health plan. HEDIS measures, set by the National Committee for Quality Assurance, provide a score or “report card” on a health plan’s effectiveness and quality.^37^ For postpartum care, HEDIS tracks the percentage of women who had a visit “on or between 7 and 84 days after delivery.”^38^ However, respondents noted that this time frame conflicts with ACOG’s 2018 recommendation for the initial assessment to occur within “the first 3 weeks postpartum.” One respondent remarked, “ACOG is saying the earlier touch bases are important, but we are still held to HEDIS metrics.” By only measuring visits between 7 and 84 days after delivery, HEDIS neglects those occurring between 0 and 6 days postpartum. Moreover, organizations “get credit” for only one HEDIS postpartum visit, whereas ACOG recommends multiple visits throughout the postpartum period. Finally, respondents noted that the HEDIS measure for postpartum care is a process measure that only accounts for whether a visit occurred, not its content nor the quality of the care provided during the visit.

Another challenge in assessing maternal health interventions is the lack of comprehensive statewide data. Respondents explained that individual insurance companies cannot track interventions for high-priority issues such as maternal mortality because their birthing populations simply are not “big enough.” Furthermore, variations in the design and implementation of interventions among organizations complicate efforts to identify those effective at scale.

### Opportunities

Although barriers persist, participants highlighted five main opportunities to improve postpartum care: restructuring postpartum visits, integrating technology, leveraging the health care workforce, aligning incentives and reimbursement, and envisioning an ideal postpartum care system (Table 3).

**Table 3.** Payors and Purchasers: Perceived Opportunities to Improve Postpartum Care

Themes	Subthemes	Exemplary quotes
Timing and frequency of visits	Dyadic visits/dyadic care	“The baby and the mother or *the baby and the birthing person are really the unit of care*. And you think about, well, how is healthcare delivered to both of them in that postpartum period? And the mom’s taking the baby to all of those like the one-week check, the two-week check … *those are opportunities where she’s having to*—with this little baby—get herself dressed and up and transport it into the office. And those are all touch points with the moms… .” (P07)
		“I think more *co-locating care* [is needed]. It’s just with separate physical locations, it does make it [visits] very difficult. I think *family physicians are very well-positioned to do it because they’re trained to perform newborn exams and to deliver babies and to take care of postpartum parents*.” (P01)
	Align HEDIS—expand HEDIS date range	“… [R]outinely, for all patients, *if we would get [HEDIS] credit for everything, then I think many of us would want to see them [patients] closer to the three weeks or even before three weeks.* I mean, because we know that if we start antidepressants or interventions for postpartum depression sooner than six weeks, it’s more successful.” (P20)
Technology	Online communities as support	“Bravo to the folks who have given birth to creating our own communities in this space. *Like the online world is so robust*. And actually, with a lot of really helpful information that I don’t think is misinformation … . This is a *space where the medical community doesn’t have the answers to a lot of what folks are living through*. And therefore, the online space is turned into a space of support … . [I] think we have to think about how do we honor and elevate and make it more *accessible to everyone*.” (P19)
	Apps/telehealth as an extension of in-person care	“I think if you could do a pap smear by telehealth, we’d have a lot better postpartum [uptake]. Because a lot of these moms, if they could do their *postpartum visit by Telehealth, they would do it*. They don’t want to drag their brand-new baby into the clinic.” (P08)
		“*I think they’re good [digital health apps], but it can’t replace the actual human*. I need to talk to you, I need to find out what you’re doing.” (P20)
		“And do you believe digital tools can be helpful in achieving comprehensive postpartum care? “Response: *Do I believe that? Yes, but not in lieu of*. You know what I mean? You can’t just say, oh yeah, like I can give you the Calm app, and you’re good.” (P20)
	Issues in tech equity	“… [S]o digital tools, there’s a lot of inequity in who gets money to build the tool. Wildflower Health is a great example. I really loved Wildflower. But Wildflower, the tool that was built with Wildflower wasn’t addressing Black women.” (P07)
Workforce	Family care physicians placed to provide dyadic care	“…it’s a space of opportunity. *We know that we have a workforce shortage*. Who is going to care for the birthing folks? Some of the *family docs* I think are [able to], especially those who practice full scope. *Med peds* is another one…because of their type of training. I think a lot of them feel comfortable doing that … *the more that you can integrate, coordinate, bridge gaps, have every touch being meaningful, the better*.” (P19)
		“And for a long time, pediatricians and family medicine—*I mean, family medicine helps bridge this because they can do the baby care, and they can do the mom care. And midwives do some of this*. They do the baby care and the mom care. But—I mean, the ideal system would be that *every time that unit, that baby birthing person unit, goes in for health care, they both get a touch in the health care system*.” (P07)
	Integrating doulas more into postbirth teams	“They’re going in and providing *support and education*. … [and] from the *communities [and] have that cultural sensitivity*.” (P07)
		“And that would be a really *good way to bring members to their postpartum visit because they have a good relationship* with the doulas. I don’t know if you’ve had that experience, but *doulas are amazing through the care for them—through the prenatal—through the delivery*. So, they have a special relationship. *So, I think that is a very good benefit, and we should all really take advantage of that*.” (P06)
Ideal systems	Home-based care	“And then actually *provide that support, reach out to them*, get a hold of them, get them to engage, have them be able to and have *enough support in their lives and even just the childcare to come in for more visits if that’s what they need, provide support at home*.” (P11)
		“… one of the things that we have talked about is doing *postpartum in-home care* and having *nurse practitioners go out and do postpartum visits* … we are implementing that for high-risk moms, [and] that is going to be one of the innovative things that we have done to achieve our postpartum visits. And how *nice for the mom to have her postpartum visit and not have to leave her home*.” (P08)
	FQHC model	“… that’s another *advantage of our FQHCs, where we have pediatricians and OBs right in the same spot*. A lot of our private docs don’t offer that. The OBs upstairs and the pediatricians downstairs or something like that. And if you have childcare in the middle, it’s ideal. If you have a *family practice physician*, you can do that …. Often, it’s the mom who has mental health issues and they *can bill under the child’s Medi-Cal number for care for the family, for the mother*. And it’s about time. Because I know when I was in practice, I worked at FQHC, and you just provided [care and billing] through the child because really the family is the unit.” (P08)
Reimbursement	Bundled payments—for postpartum	“You really should *pay people separately above and beyond what you pay them for doing the delivery for seeing patients several times in the postpartum period*… See, the whole issue is that we’re still laboring under the global OB concept whereas part of the global fee everything you’re basically paid for everything that happens during the first six weeks … . *We really should make that a separately reimbursable period of time and extend it beyond six weeks*. I mean, this concept that somehow at six weeks all mothers are magically healthy is—I mean, it was never based on anything.” (P09)
	Billing for dyadic care or dyad as a unit for billing	“...[u]ltimately, of course, what we really should be doing is… *migrating to a system of bundled care where it’s just one price that includes antenatal care, delivery, and then a year of well women care, and whatever, afterward then that would make a lot more sense.* Probably like a two-year bundle … . [I]’ve always advocated for including also care of the newborn for the first month after delivery in the bundle because you incentivize people.” (P09)

FQHC, Federally Qualified Health Center.

#### Restructuring postpartum visits

Insurance leaders proposed two strategies to improve coordination of care and the timing and frequency of postpartum visits: adopting mother–infant dyadic care models and aligning HEDIS measures with ACOG guidelines.

Multiple (five of nine) respondents recommended dyadic care, which integrates newborn and maternal care into a single visit. The newborn and birthing person becomes a “unit of care,” with each newborn visit offering an additional “touchpoint” to address maternal health needs. Currently, newborns are seen by pediatricians in the first week(s) postbirth to assess weight gain, feeding, and other emergent issues.^39^ Meanwhile, mothers typically have postpartum visits weeks later, often with different providers at separate locations. Dyadic care can ease the burden on new parents managing transportation and the physical strain of attending multiple appointments. One respondent emphasized scheduling earlier postpartum visits, particularly to address mental health issues: “We know if we start antidepressants or interventions for postpartum depression sooner … [i]ts more successful.” Dyadic care could support this recommendation since newborns “are being seen [early] in pediatrics.”

#### Technological integration

Participants described digital health tools as crucial for making postpartum care accessible. Respondents described text messages and phone applications as particularly effective for patient education and appointment reminders. They highlighted the convenience of digital health tools and virtual visits as particularly valuable during the postpartum period: “a lot of these moms, if they could do their postpartum visit by telehealth, they would do it. They don’t want to drag their brand-new baby into the clinic.” However, participants cautioned that digital tools must undergo equity and accessibility assessments before widespread implementation. One warned that “inequities in who gets the money” for app development often reflect “who is building it,” leading to apps that primarily target white, upper-middle-class populations rather than marginalized groups. Another interviewee urged that technology should complement care, not “replace an actual human.”

#### Leveraging the health care workforce

Shortages in the obstetric workforce limit the availability of postpartum services,^35,40^ prompting several interviewees to suggest integrating internal medicine–pediatrics (med-peds) and family practice physicians into postpartum care. These physicians, experienced with both newborns and postpartum parents, are well-positioned to address postpartum and pediatric issues simultaneously. One respondent stated, “the ideal system would be that every time that unit—the baby and birthing person—goes in for healthcare, they both get touched in the healthcare system.”

Respondents also emphasized the importance of doulas and community midwives in connecting postpartum people of color to community services. In California, doulas are particularly active as Medi-Cal has covered their services since January 1, 2023.^26^ One insurance provider highlighted the “special relationship” doulas share with their patients, founded on “cultural sensitivity” and trust, especially within marginalized communities. Participants noted that CHWs and community midwives help bridge the gap for patients who may distrust traditional medical systems.

#### Aligning incentives and reimbursement

Two main suggestions for updating postpartum billing and reimbursement emerged from the interviews: separate bundled payments for the postpartum period and billing for dyadic visits. A respondent from a commercial payor suggested “separating” postpartum payments from the “global OB concept [bundle]” and argued that postpartum care should be “separately reimbursable and extend beyond the 6-week period,” emphasizing “the concept that mothers should be healed at 6 weeks after birth was never built on anything.” Another respondent recommended separating pregnancy care from postpartum care and suggested that the postpartum bundle should cover “*two years*” to adequately meet postpartum needs. They also stressed that including newborn coverage in these packages could further incentivize clinicians and patients to provide and attend postpartum visits throughout the entire postpartum period.

#### Ideal system

Each interview included a question about respondents’ views on an ideal postpartum system. Several interviewees described FQHCs as an “ideal model” for postpartum care, citing their wraparound services—multiple services available at one location—that enable both newborn and mother to be seen in the same visit. The convenience of this approach may increase timely postpartum visits. At-home visits by nurse practitioners, doulas, or other CHWs were also presented as key components of an ideal system, helping to reduce barriers such as transportation, newborn exposure to illnesses, and the need for multiple clinical visits.

## Discussion

This study presents a novel examination of insurance payors’ and insurance providers’ perspectives on postpartum barriers and opportunities, a perspective that remains understudied. We found widespread consensus that California’s current postpartum care system fails to adequately address patients’ needs, with significant barriers hindering the implementation of comprehensive postpartum care.

First, the literature identifies low visit attendance as a persistent issue in postpartum care.^12^ Patients face structural challenges such as “delayed insurance enrollment, provider scarcity, and care interruptions,”^12^ whereas clinicians identify the “complexity of obtaining and maintaining insurance” as barriers to attending visits.^16^ Our respondents underscore low reimbursement rates as an important barrier, consistent with studies showing that higher Medicaid reimbursement rates correlate with increased prenatal visit attendance.^41^ Our study also identifies global maternity fees as a concern: bundling postpartum care within larger maternal care packages may disincentivize postpartum visits if funds are exhausted during the perinatal period. This model creates a “perverse incentive” that discourages scheduling postpartum visits,^23,42^ with one respondent noting, “the best patient was the one who didn’t show up.” Respondents propose separately bundling postpartum care payments for the first year after birth—a strategy previously implemented by Medicaid and commercial plans for prenatal care—that could be repurposed for postpartum care.^43,44^

Second, our findings reveal a disconnect between current professional recommendations and HEDIS measures for postpartum care; ACOG recommends care “within the first three weeks” and “ongoing” care through the first 12 weeks postpartum,^7^ whereas HEDIS credits only one postpartum visit between 7 and 84 days, excluding visits in the first week and failing to incentive additional “ongoing” visits for postpartum issues. Revising HEDIS requirements to better align with professional recommendations and address the content of care could improve both adherence to professional recommendations and quality of care. For example, in 2020, HEDIS added a requirement for postpartum depression screenings to be conducted by the 60-day postpartum mark, which correlates with a rise in postpartum depression screenings and follow-ups over the past 3 years.^45^

Furthermore, participants highlight concerns about fragmentation between postpartum and primary care, mirroring patient and clinician challenges with referrals and ambiguous clinician roles,^15^ contributing to a lack of clinician continuity.^16^ To address these challenges, they recommend adopting a dyadic care model and leveraging the existing health care workforce to address obstetric shortages, ideas supported by data from low-income postpartum women and clinicians.^17^ Interviewees note that family medicine providers are “well-positioned” for dyadic care due to their training in treating both newborns and adults and their existing billing systems and facilities. In practice, the dyadic model has demonstrated positive outcomes, including higher rates of maternal mental health screenings and newborn immunizations using the two-generation model,^46^ and improved attendance and monitoring of issues such as hypertensive disorders, postpartum depression, and feeding challenges.^47^

Participant views that integrating telemedicine, home visits, and doulas could improve postpartum care aligns with existing literature, showing that home visits improve health outcomes for parents and children and increase maternal satisfaction.^14,17,48,49^ Although participants cautioned against over-reliance on doulas, they recognized their unique role in reaching communities of color, a finding supported by the literature.^50^

Last, participants identified FQHCs as an “ideal model” for comprehensive postpartum care that is already in use in California. These centers offer co-located wraparound services for both newborns and birthing parents^51,52^ and are well-integrated into their respective communities, enabling them to provide nuanced, culturally sensitive care—a critical need given the systemic inequalities in maternal health across the United States.

These insights are particularly relevant as California undergoes a maternal health transformation. The Department of Health Care Services (DHCS) Birthing Care Pathway aims to modernize postpartum care, but expecting clinicians to do more with limited resources presents a challenge. A recent survey found that California obstetrician/gynecologists and midwives already feel constrained by limited resources and time, but prioritize meeting measures such as HEDIS.^21^ Since most health plans adhere to national HEDIS measures that account for whether a visit occurs, modifying these measures is one avenue to align incentives to encourage ongoing and comprehensive postpartum care. Other suggestions offered by payors, such as co-located and dyadic care models, might support clinicians to implement earlier initiation and increase continuity of care, and a dedicated postpartum payment bundle may facilitate higher payments and increase visit attendance.^21^

This study’s strengths include capturing perspectives from key insurance stakeholders and offering a comprehensive view of postpartum care delivery, including barriers and opportunities for improvement. The qualitative approach provides in-depth insights from a significant insurance market share across California. The small sample size limits generalizability; however, the participants’ organizations cover a large number of Californians. Recruitment in this population of upper-level administrators was challenging due to concerns of employer confidentiality, competition, and schedule conflicts, despite direct email contact through professional connections. A further limitation is the focus on payors and purchasers, potentially overlooking the experiences of frontline clinicians and patients directly impacted by standards and procedures. However, some participants were current or former providers or were parents themselves and could offer insights from those perspectives. Moreover, findings from this study support existing literature on clinicians’ and patients’ views, and the unique insights from payors’ perspectives in California provide an important complement to the literature.

## Conclusions

This study addresses a gap in perinatal literature by examining insurers’ perspectives on improving postpartum care in California. Participants identified issues such as low reimbursement rates, fragmented care, and misaligned quality measures as key; they emphasized the need for solutions such as revised payment models, aligning HEDIS measures with professional recommendations, adoption of dyadic care models, and integrating doulas, CHWs, and technology into the care system. Ultimately, coordination among payors, patients, and providers is essential for the successful implementation and sustainability of these policies. This study fills a critical gap in the literature, offering valuable insights into payors’ perspectives and fostering a more comprehensive discussion of the fourth trimester.

## Abbreviations Used

ACOG: American College of Obstetrics and Gynecology
CDC: Centers for Disease Control and Prevention
CHBRP: California health Benefits Review Program
CHW: Community healthcare worker
DHCS: Department of Healthcare Services
FQHC: Federally Qualified Health Center
HEDIS: Healthcare Effectiveness Data and Information Set
OB: Obstetric
